# Deep learning and radiomics framework for PSMA-RADS classification of prostate cancer on PSMA PET

**DOI:** 10.1186/s13550-022-00948-1

**Published:** 2022-12-29

**Authors:** Kevin H. Leung, Steven P. Rowe, Jeffrey P. Leal, Saeed Ashrafinia, Mohammad S. Sadaghiani, Hyun Woo Chung, Pejman Dalaie, Rima Tulbah, Yafu Yin, Ryan VanDenBerg, Rudolf A. Werner, Kenneth J. Pienta, Michael A. Gorin, Yong Du, Martin G. Pomper

**Affiliations:** 1 Department of Biomedical Engineering, Johns Hopkins University School of Medicine, 601 N Caroline St. JHOC 4263, Baltimore, MD 21287 USA; 2 The Russell H. Morgan Department of Radiology and Radiological Science, Johns Hopkins University School of Medicine, Baltimore, MD USA; 3 The James Buchanan Brady Urological Institute and Department of Urology, Johns Hopkins University School of Medicine, Baltimore, MD USA; 4 Department of Nuclear Medicine, Konkuk University Medical Center, Konkuk University School of Medicine, Seoul, Korea; 5 Department of Nuclear Medicine, Xinhua Hospital, Shanghai Jiao Tong University School of Medicine, Shanghai, China; 6 Department of Nuclear Medicine, University Hospital Würzburg, Würzburg, Germany; 7 The Milton and Carroll Petrie Department of Urology, Icahn School of Medicine at Mount Sinai, New York, NY USA

**Keywords:** PSMA-RADS, PSMA PET, Deep learning, Classification, t-SNE, Prostate cancer

## Abstract

**Background:**

Accurate classification of sites of interest on prostate-specific membrane antigen (PSMA) positron emission tomography (PET) images is an important diagnostic requirement for the differentiation of prostate cancer (PCa) from foci of physiologic uptake. We developed a deep learning and radiomics framework to perform lesion-level and patient-level classification on PSMA PET images of patients with PCa.

**Methods:**

This was an IRB-approved, HIPAA-compliant, retrospective study. Lesions on [^18^F]DCFPyL PET/CT scans were assigned to PSMA reporting and data system (PSMA-RADS) categories and randomly partitioned into training, validation, and test sets. The framework extracted image features, radiomic features, and tissue type information from a cropped PET image slice containing a lesion and performed PSMA-RADS and PCa classification. Performance was evaluated by assessing the area under the receiver operating characteristic curve (AUROC). A t-distributed stochastic neighbor embedding (t-SNE) analysis was performed. Confidence and probability scores were measured. Statistical significance was determined using a two-tailed *t *test.

**Results:**

PSMA PET scans from 267 men with PCa had 3794 lesions assigned to PSMA-RADS categories. The framework yielded AUROC values of 0.87 and 0.90 for lesion-level and patient-level PSMA-RADS classification, respectively, on the test set. The framework yielded AUROC values of 0.92 and 0.85 for lesion-level and patient-level PCa classification, respectively, on the test set. A t-SNE analysis revealed learned relationships between the PSMA-RADS categories and disease findings. Mean confidence scores reflected the expected accuracy and were significantly higher for correct predictions than for incorrect predictions (*P* < 0.05). Measured probability scores reflected the likelihood of PCa consistent with the PSMA-RADS framework.

**Conclusion:**

The framework provided lesion-level and patient-level PSMA-RADS and PCa classification on PSMA PET images. The framework was interpretable and provided confidence and probability scores that may assist physicians in making more informed clinical decisions.

**Supplementary Information:**

The online version contains supplementary material available at 10.1186/s13550-022-00948-1.

## Background

Prostate cancer (PCa) is one of the most common cancers and a leading cause of cancer-related death in men [1]. There has been an increasing interest in positron emission tomography (PET) agents targeting prostate-specific membrane antigen (PSMA), a transmembrane protein overexpressed on PCa cells, for imaging and directing therapy of PCa [2]. Radiotracer-avid and non-avid pitfalls have been described with PSMA PET imaging [3, 4]. Reliable classification of lesions with or without radiotracer uptake is an important clinical step in verifying the detection and determining the prognosis of PCa [5]. We developed a PSMA reporting and data system (PSMA-RADS version 1.0) framework to classify PSMA PET scans and individual findings that reflect the probability of PCa, thereby guiding management [5, 6]. We organized the PSMA-RADS framework around a 5-point scale where a higher score indicates a greater likelihood of PCa [5].

While medical images are typically visually evaluated by trained radiologists, this process may be time-consuming and subject to operator variability [7]. Radiomics is a rapidly advancing field that aims to perform high-throughput extraction of clinically relevant features from radiologic data to build diagnostic and prognostic models [8, 9]. Unlike traditional radiomics workflows that utilize engineered handcrafted features, deep learning (DL) approaches can automatically extract deep features to directly model medical endpoints from the input images [9]. Automated artificial intelligence and DL methods have significant advantages over manual evaluation, including more consistent extraction of radiomic features and reliable characterization of disease [7]. Several machine learning and DL applications have been developed for PSMA PET in patients with metastatic disease, including radiomics-based risk stratification, attenuation map estimation for PSMA PET/magnetic resonance imaging (MRI), and bone and lymph node lesion detection in PSMA PET/computed tomography (CT) images [10–13].

While DL methods can be conveniently treated as a black box, deep neural networks often suffer from a lack of interpretability [14]. Despite having improved levels of accuracy in recent years, modern neural networks are not well calibrated and tend to be overconfident in their predictions [15]. Reliable confidence estimates and likelihood measures are important for the interpretability of DL methods and could assist physicians in facilitating clinical decisions [15].

We developed an interpretable framework that incorporates both DL and radiomics for automated PSMA-RADS and PCa classification on PSMA PET images. The framework provided both lesion-level and patient-level predictions as well as calibrated confidence scores that reflected the level of certainty for those predictions and probability scores that reflected the likelihood of PCa. A t-distributed stochastic neighbor embedding (t-SNE) analysis provided insight into learned relationships between the PSMA-RADS categories and disease findings on PSMA PET.

## Materials and methods

### PSMA PET/CT dataset

**Fig. 1:**
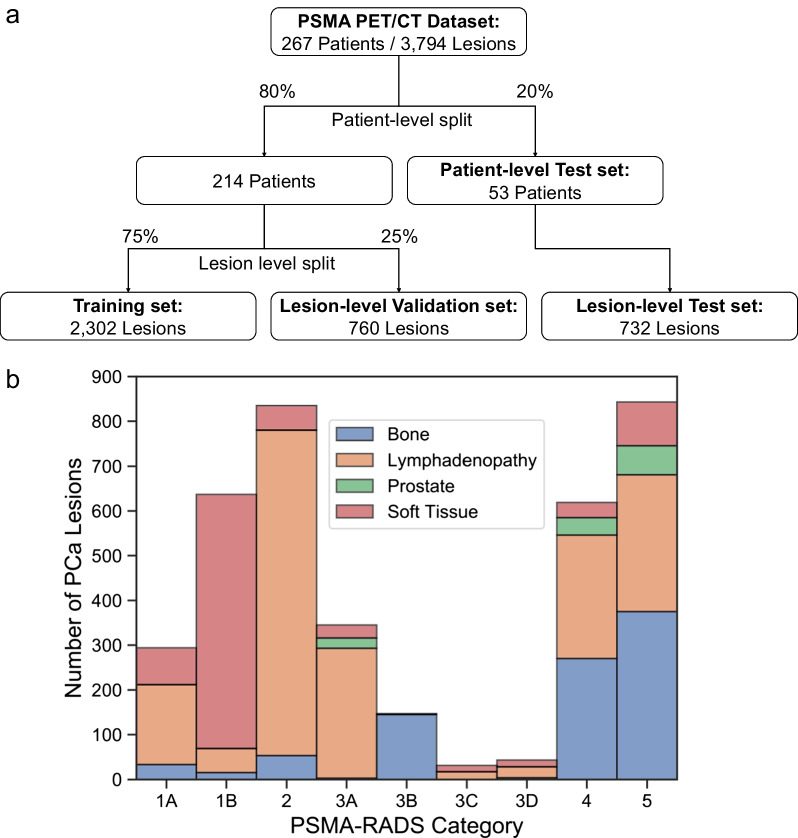
Flowchart of data partitioning (**a**) and a histogram of dataset characteristics (**b**), including the distribution of PSMA-RADS categories and tissue type information

This was an IRB-approved, HIPAA-compliant, retrospective study. The data consisted of 267 [^18^F]DCFPyL PET/CT scans acquired at 60 min post-injection across two different scanners (Table 1). Four trained nuclear medicine physicians manually segmented 3794 lesions on a per-slice basis in the axial view. Each lesion was assigned to one of the nine PSMA-RADS categories, and specific anatomic locations were recorded [16]. While each lesion was annotated by a single physician, the PSMA-RADS framework has high inter-observer agreement across readers with varying experience levels [17, 18]. The observed PSMA-RADS categories were used as ground truth. The data were randomly partitioned into training, validation, and test datasets with 2302, 760, and 732 lesions, respectively (Fig. 1a). Data from 53 patients were randomly partitioned into the patient-level test set. The remaining data were split into lesion-level training and validation sets. The framework was evaluated considering both in- and out-of-patient distributions [19]. The dataset characteristics are described in Table 1 and Fig. 1b.

**Table 1:** Dataset characteristics

	Lesion-level distribution			
	Training set	Validation set	Test set	Total
Number of lesions	2302	760	732	3794
GE Discovery RX^a^	673	221	129	1023
Siemens Biograph mCT^b^	1629	539	603	2771
PSMA-RADS-1A	149	36	109	294
PSMA-RADS-1B	382	129	126	637
PSMA-RADS-2	442	165	228	835
PSMA-RADS-3A	225	57	63	345
PSMA-RADS-3B	81	32	34	147
PSMA-RADS-3C	19	5	7	31
PSMA-RADS-3D	15	6	22	43
PSMA-RADS-4	446	139	34	619
PSMA-RADS-5	543	191	109	843

	Patient-level distribution		
	Training + Validation sets	Test set	Total
Number of patients	214	53	267
GE Discovery RX^a^	79	19	98
Siemens Biograph mCT^b^	135	34	169
PSMA-RADS-1A	3	–	3
PSMA-RADS-1B	2	3	5
PSMA-RADS-2	21	6	27
PSMA-RADS-3A	15	5	20
PSMA-RADS-3B	12	2	14
PSMA-RADS-3C	6	–	6
PSMA-RADS-3D	–	1	1
PSMA-RADS-4	33	5	38
PSMA-RADS-5	122	31	153

^a^GE Healthcare, Waukesha, WI, USA

^b^Siemens Healthineers, Erlangen, Germany

### DL and radiomics framework

**Fig. 2:**
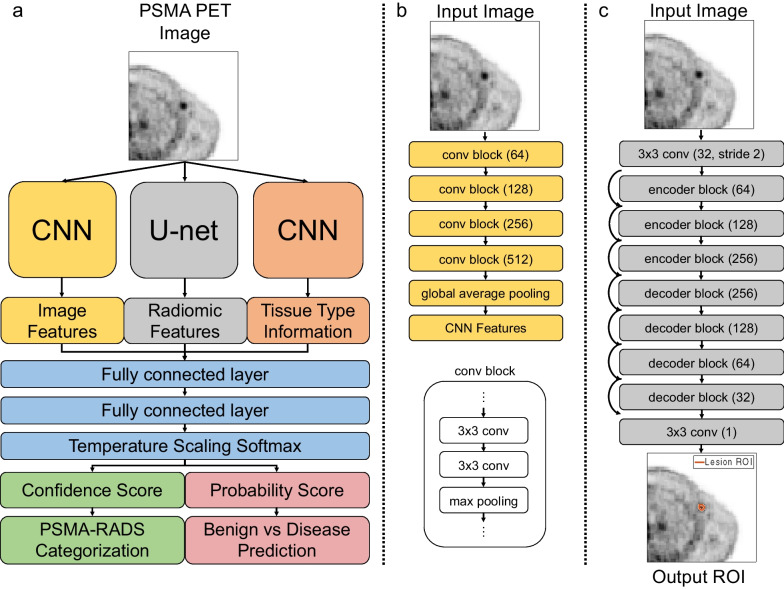
Deep learning and radiomics framework (**a**). CNN architecture (**b**). U-net architecture where curved arrows represent residual connections (**c**). Values in parentheses refer to the feature map depth

A framework was developed using DL and radiomics to perform PSMA-RADS and PCa classification of lesions on PSMA PET images (Fig. 2). A deep convolutional neural network (CNN) extracted image features from a cropped PET image slice containing a lesion to implicitly capture local contextual and global information. Image slices were cropped by a bounding box with a diagonal length of 7.5 times the lesion diameter (Additional file 1: Fig. S1). The lesion was placed at the center of the region of interest (ROI) to classify a single lesion while avoiding confusion with other lesions. A U-net delineated the lesion ROI on the cropped image slice (Additional file 1: Figs. S2–S3 and Table S1) [20–22]. Radiomic features explicitly captured intensity and morphology characteristics from lesion ROIs. Since the PSMA-RADS framework incorporates tissue type information at the site of uptake, the lesion tissue type was extracted by a separate CNN (Additional file 1: Fig. S4 and Table S2). Tissue type information was categorized into 4 broad categories, including bone, prostate, soft tissue, and lymphadenopathy, and encoded into one-hot vectors [23]. The extracted features were passed into a fully connected network to yield softmax probabilities indicating the likelihood of belonging to one of the nine PSMA-RADS categories [24].

The framework was trained on the training set by minimizing a class-weighted categorical cross-entropy loss function via the adaptive moment estimation stochastic optimization algorithm [25]. The framework was trained on cropped image slices to augment the data with a batch size of 512 samples for 500 epochs. Hold-out cross-validation during hyperparameter optimization and early stopping was applied to a randomly partitioned hold-out set consisting of 15% of the training dataset to prevent overfitting. See the Supplementary Information for data processing and network architecture details.

### PSMA-RADS classification

The framework performed per-lesion and per-patient PSMA-RADS classification. Softmax probabilities were averaged across slices for lesion-level predictions [26]. Patient-level predictions were performed by taking the highest PSMA-RADS score across all lesions on the scan following the recommended guidelines for PSMA-RADS interpretation [5]. Lesion-level performance was evaluated on the validation and test sets. Patient-level performance was evaluated on the test set. The receiver operating characteristic (ROC) curve, area under the ROC curve (AUROC), confusion matrix, overall accuracy, precision, recall, and F1 score were assessed. Accuracy metrics were class-weighted, and ROC curves were micro-averaged. The framework’s performance was evaluated when using both the physician-annotated and automatically extracted radiomic feature and tissue type information inputs. Performance was compared across different scanners.

### PCa classification

The framework provided a broad PCa classification, formulated as a binary classification task, based on the likelihood of benign versus disease findings according to the PSMA-RADS framework [5]. PSMA-RADS-1 and -2 lesions were categorized as likely benign findings, and PSMA-RADS-3, 4, and 5 lesions were categorized as likely disease [5]. The predicted softmax probabilities were summed across the respective PSMA-RADS categories. Lesion-level and patient-level performance was evaluated on the validation and test sets when using manually and automatically extracted inputs and compared across different scanners.

### t-SNE analysis

The framework’s predictions were visualized using t-SNE to provide an understanding of how the framework clusters its predictions. t-SNE is an unsupervised dimensionality reduction technique used to visualize the local structure and global geometry of high-dimensional data [27]. The framework’s predictions were visualized in two dimensions via t-SNE with principal components analysis initialization.

### A confidence score for PSMA-RADS classification

The framework provided confidence scores reflecting the expected level of accuracy. Temperature scaling, a single-parameter variant of Platt scaling, was performed to calibrate the framework’s outputs before the softmax activation [15]. The optimal temperature, *T*, for temperature scaling calibration was found on the validation set and applied on the test set to yield well-calibrated softmax probabilities. Confidence scores were defined as the calibrated softmax probability corresponding to the predicted PSMA-RADS category. Confidence histograms were observed, and confidence scores of accurate and inaccurate predictions were compared. Confidence scores were visualized on t-SNE space.

### A probability score for PCa

The framework provided a probability score that reflected the likelihood of PCa. The probability scores were derived by summing the calibrated softmax probabilities across the respective PSMA-RADS categories corresponding to disease findings. The distribution of probability scores for the test set predictions was compared on boxplots according to their PSMA-RADS categories and visualized on a t-SNE scatter plot.

### Feature importance

Feature importance experiments were performed to evaluate the robustness of the framework. Different input combinations, including the cropped PET image (I), the extracted radiomic features (F), and the tissue type of the lesion (L), were used to train the framework (Additional file 1: Table S3). The framework was evaluated on the validation set for lesion-level prediction using the manually extracted inputs for each input combination. Feature ablation experiments were also performed to further assess the importance of the radiomic features where individual radiomic features were removed from the inputs during prediction. The relative performance reductions in overall accuracy due to feature ablation compared to the model predictions without feature ablation were assessed on the validation set for lesion-level prediction using the manually extracted inputs.

### Statistical analysis

Statistical significance was determined using a two-tailed t test where a *P* < 0.05 was used to infer a significant difference. ROC curve 95% confidence and tolerance intervals were computed with 1000 bootstrap samples. Statistical analysis and data processing were implemented in Python 3.8.8 and MATLAB 2022b. The framework was implemented in TensorFlow 2.4.1 and Keras 2.4.3 on NVIDIA Quadro P5000 and NVIDIA A6000 GPUs with Linux CentOS 7.6 and Windows 10 operating systems.

## Results

### Characterizing the PSMA PET data

A histogram of the PSMA-RADS categories and tissue types across all lesions is shown in Fig. 1b. Lesion-level and patient-level distributions of the PSMA-RADS categories and scanner types are shown in Table 1. There were 898, 1873, 127, and 896 lesions with a tissue type of bone, lymphadenopathy, prostate, and soft tissue, respectively.

### PSMA-RADS classification

**Fig. 3:**
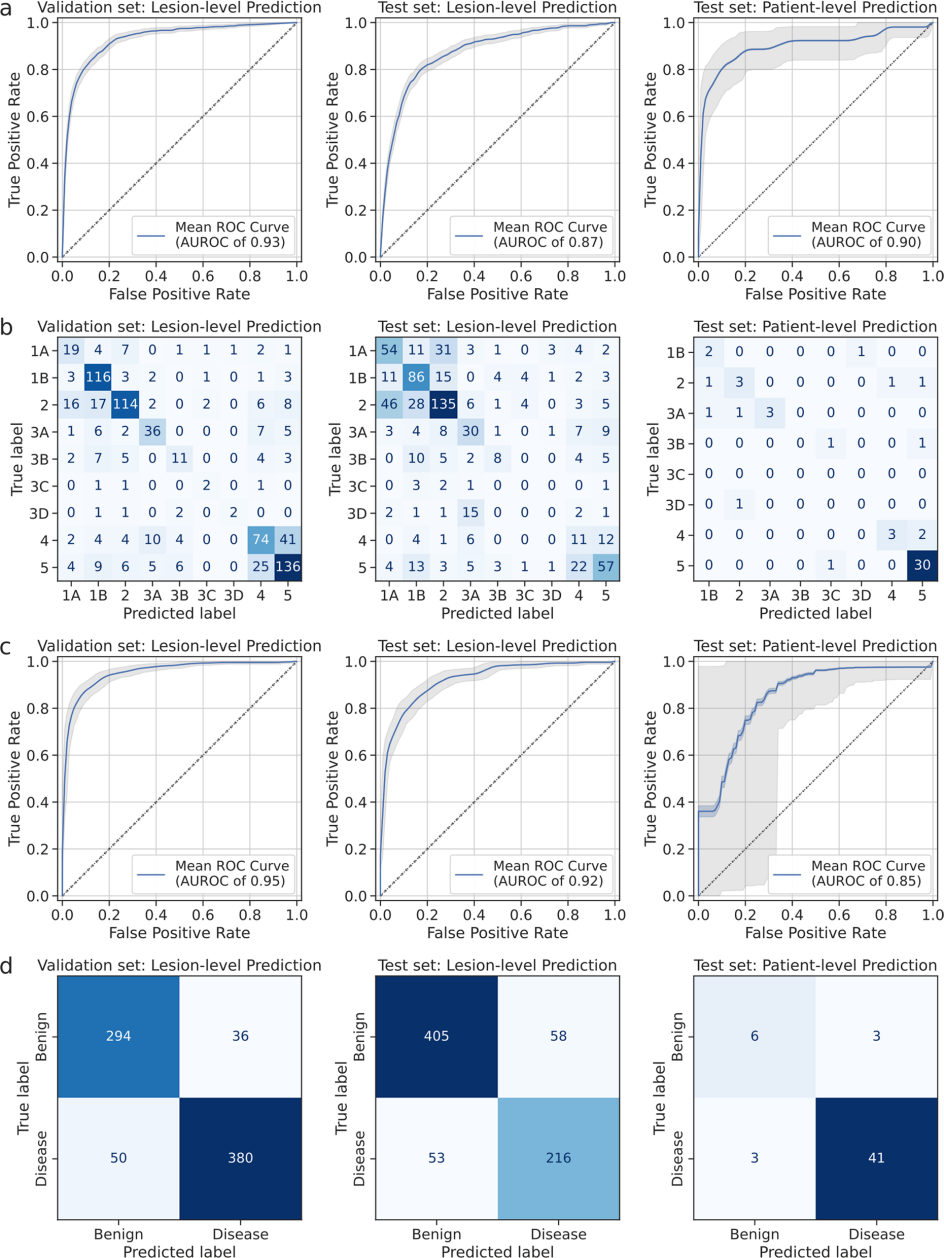
ROC curves and confusion matrices for the PSMA-RADS classification task (**a**, **b**) and the broad prostate cancer classification task (**c**, **d**) when using the automatically extracted inputs. The shaded blue and gray areas correspond to the 95% confidence intervals and the 95% tolerance intervals on the ROC curves, respectively

Accuracy metrics, ROC curves, and confusion matrices on the framework’s performance on the PSMA-RADS classification task are given in Table 2 and Fig. 3a, b. When using automatically extracted inputs, the framework yielded AUROC values of 0.93 and 0.87 (Fig. 3a) and overall accuracies of 0.67 and 0.52 on the validation and test sets, respectively, for lesion-level prediction. ROC curves and AUROC values on the validation and test sets for lesions with a tissue type of bone, lymphadenopathy, prostate, and soft tissue, respectively, are shown in Additional file 1: Fig. S5 for lesion-level prediction when using manually extracted inputs. For patient-level prediction, the framework yielded AUROC values of 0.91 and 0.90 and overall accuracies of 0.77 and 0.77 on the test set with the manually and automatically extracted inputs, respectively (Table 2, Fig. 3a). The framework’s lesion-level and patient-level overall accuracy was not significantly different across different scanners (*P* > 0.05).

**Table 2:** Performance on PSMA-RADS classification

Inputs	AUROC	Accuracy	Precision	Recall	F1 score
Validation set: Lesion-level performance					
Manual	0.95 (0.95, 0.95)	0.71 (0.68, 0.74)	0.71	0.71	0.71
Predicted RF	0.95 (0.95, 0.95)	0.70 (0.67, 0.73)	0.71	0.70	0.70
Predicted TT	0.93 (0.93, 0.93)	0.68 (0.64, 0.71)	0.67	0.68	0.67
Predicted RF + TT	0.93 (0.93, 0.93)	0.67 (0.64, 0.70)	0.67	0.67	0.67
Test set: Lesion-level performance					
Manual	0.91 (0.91, 0.91)	0.61 (0.58, 0.65)	0.62	0.61	0.61
Predicted RF	0.90 (0.90, 0.90)	0.56 (0.53, 0.60)	0.58	0.56	0.57
Predicted TT	0.88 (0.88, 0.88)	0.55 (0.52, 0.59)	0.56	0.55	0.55
Predicted RF + TT	0.87 (0.87, 0.88)	0.52 (0.48, 0.56)	0.53	0.52	0.52
Test set: Patient-level performance					
Manual	0.91 (0.90, 0.91)	0.77 (0.66, 0.89)	0.79	0.77	0.76
Predicted RF	0.87 (0.87, 0.88)	0.68 (0.55, 0.80)	0.69	0.68	0.68
Predicted TT	0.92 (0.92, 0.92)	0.81 (0.71, 0.92)	0.85	0.81	0.82
Predicted RF + TT	0.90 (0.90, 0.90)	0.77 (0.66, 0.89)	0.78	0.77	0.77

Data in parenthesis correspond to 95% confidence intervals. Manual refers to using the radiomic features extracted from manual segmentations and the manually annotated tissue types as inputs. Predicted refers to using the automatically extracted radiomic features and the automatically predicted tissue types as inputs. AUROC = area under the receiver operating characteristic. RF = radiomic features. TT = tissue types

### PCa classification

Accuracy metrics, ROC curves, and confusion matrices on the framework’s performance for PCa classification are given in Table 3 and Fig. 3c, d. The framework yielded AUROC values of 0.98 and 0.96 and overall accuracies of 0.94 and 0.89 on the validation and test sets, respectively, for lesion-level prediction using manually extracted inputs (Table 3). When using automatically extracted inputs, the framework yielded AUROC values of 0.95 and 0.92 and overall accuracies of 0.89 and 0.85 on the validation and test sets, respectively (Fig. 3c). For patient-level prediction, the framework yielded overall accuracies of 0.92 and 0.89 and AUROC values of 0.84 and 0.85, when using the manually and automatically extracted inputs, respectively, on the test set. The framework’s lesion-level and patient-level overall accuracy was not significantly different across scanners (*P* > 0.05).

**Table 3:** Performance on PCa classification

Inputs	AUROC	Accuracy	Precision	Recall	F1 score
Validation set: Lesion-level performance					
Manual	0.98 (0.98, 0.98)	0.94 (0.92, 0.95)	0.95	0.94	0.94
Predicted RF	0.98 (0.98, 0.98)	0.93 (0.91, 0.95)	0.94	0.93	0.94
Predicted TT	0.95 (0.95, 0.95)	0.89 (0.87, 0.91)	0.91	0.89	0.90
Predicted RF + TT	0.95 (0.95, 0.95)	0.89 (0.86, 0.91)	0.91	0.88	0.90
Test set: Lesion-level performance					
Manual	0.96 (0.96, 0.96)	0.89 (0.87, 0.92)	0.81	0.94	0.87
Predicted RF	0.96 (0.96, 0.96)	0.89 (0.87, 0.91)	0.80	0.93	0.86
Predicted TT	0.92 (0.92, 0.92)	0.85 (0.83, 0.88)	0.79	0.81	0.80
Predicted RF + TT	0.92 (0.92, 0.92)	0.85 (0.82, 0.87)	0.79	0.80	0.80
Test set: Patient-level performance					
Manual	0.84 (0.84, 0.85)	0.92 (0.85, 1.00)	0.93	0.98	0.96
Predicted RF	0.88 (0.87, 0.88)	0.92 (0.85, 1.00)	0.93	0.98	0.96
Predicted TT	0.84 (0.84, 0.85)	0.89 (0.80, 0.97)	0.93	0.93	0.93
Predicted RF + TT	0.85 (0.84, 0.86)	0.89 (0.80, 0.97)	0.93	0.93	0.93

Data in parentheses correspond to 95% confidence intervals. Manual refers to using the radiomic features extracted from manual segmentations and the manually annotated tissue types as inputs. Predicted refers to using the automatically extracted radiomic features and the automatically predicted tissue types as inputs. AUROC = area under the receiver operating characteristic. RF = radiomic features. TT = tissue types

### t-SNE analysis

**Fig. 4:**
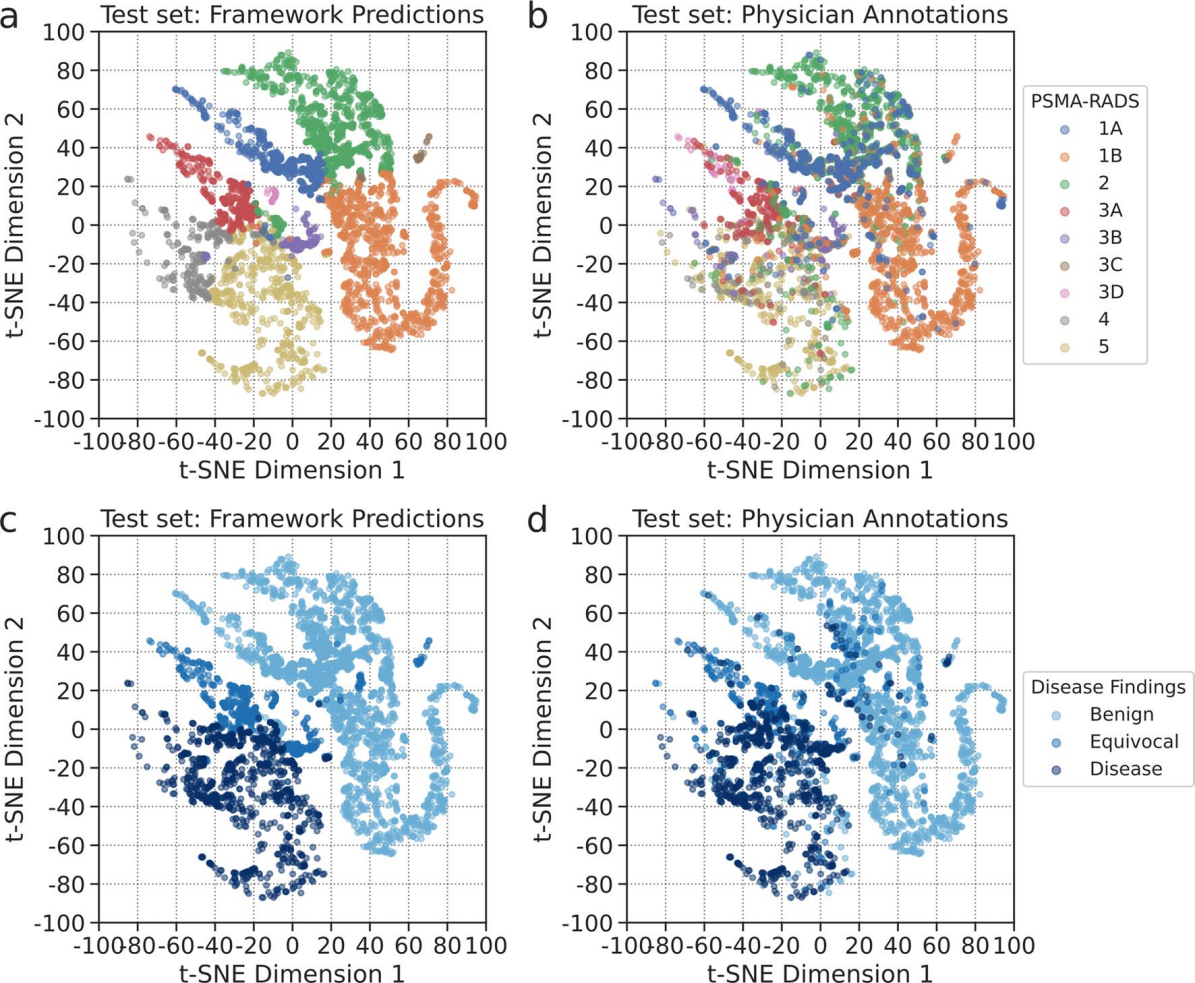
t-SNE scatter plots of predictions on test set labeled according to their predicted PSMA-RADS categories (**a**, **b**) and to their predicted PSMA-RADS categories corresponding to benign, equivocal, and disease findings (**c**, **d**)

The t-SNE scatter plots of the framework’s predictions are shown in Fig. 4. The framework formed well-defined clusters of the predicted PSMA-RADS categories (Fig. 4a). These clusters were preserved when labeled according to the physician annotations (Fig. 4b). The framework learned the global relationship between benign, equivocal, and disease findings (Fig. 4c, d). PSMA-RADS-1A, -1B, and -2 predictions were clustered together in the upper right of the t-SNE space forming a global cluster of benign findings. PSMA-RADS-4 and -5 predictions, findings that were highly likely PCa, were closely clustered in the lower left of the t-SNE space. Equivocal findings corresponding to PSMA-RADS-3A, -3B, and -3D predictions were closely clustered near the PSMA-RADS-4 and -5 predictions at the center of the t-SNE space between the global benign and disease clusters (Fig. 4a). This reflects the uncertainty of those equivocal findings on their compatibility with PCa. Interestingly, PSMA-RADS-3C predictions were clustered near PSMA-RADS-1B and -2 predictions (Fig. 4a). This may be because PSMA-RADS-3C findings are atypical for PCa and likely to be other non-prostate malignancies or benign tumors [5].

### A confidence score for PSMA-RADS classification

**Fig. 5:**
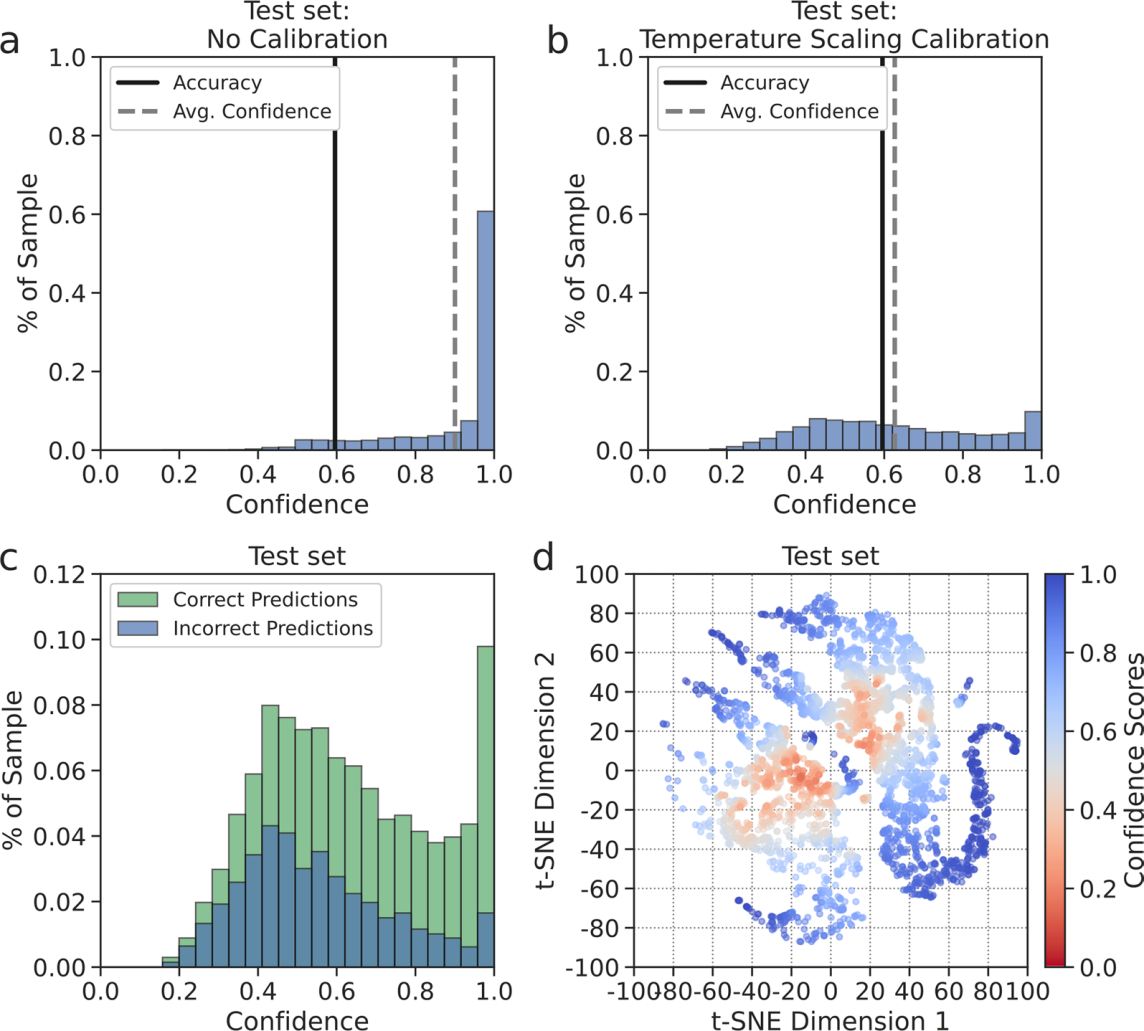
Confidence histograms comparing the average confidence to expected accuracy (**a**, **b**). Stacked confidence histogram for correct and incorrect predictions (**c**). Confidence scores were depicted on a t-SNE scatter plot (**d**)

The optimal temperature for temperature scaling was *T* = 4.26. Confidence histograms before and after performing temperature scaling calibration are shown in Fig. 5a, b. Before calibration, the framework’s average confidence was 0.90 on the test set. After calibration, the average confidence was 0.63 reflecting the framework’s overall accuracy of 0.61. A confidence histogram comparing correct and incorrect predictions is shown in Fig. 5c. The mean confidence scores were significantly higher (*P* < 0.05) for correct predictions (0.68) than for incorrect predictions (0.55). The distribution of confidence scores on t-SNE space is shown in Fig. 5d. The framework was less confident of predictions near the boundaries between individual PSMA-RADS subcategory clusters and more confident of predictions farther away from those boundaries.

### A probability score for PCa

**Fig. 6:**
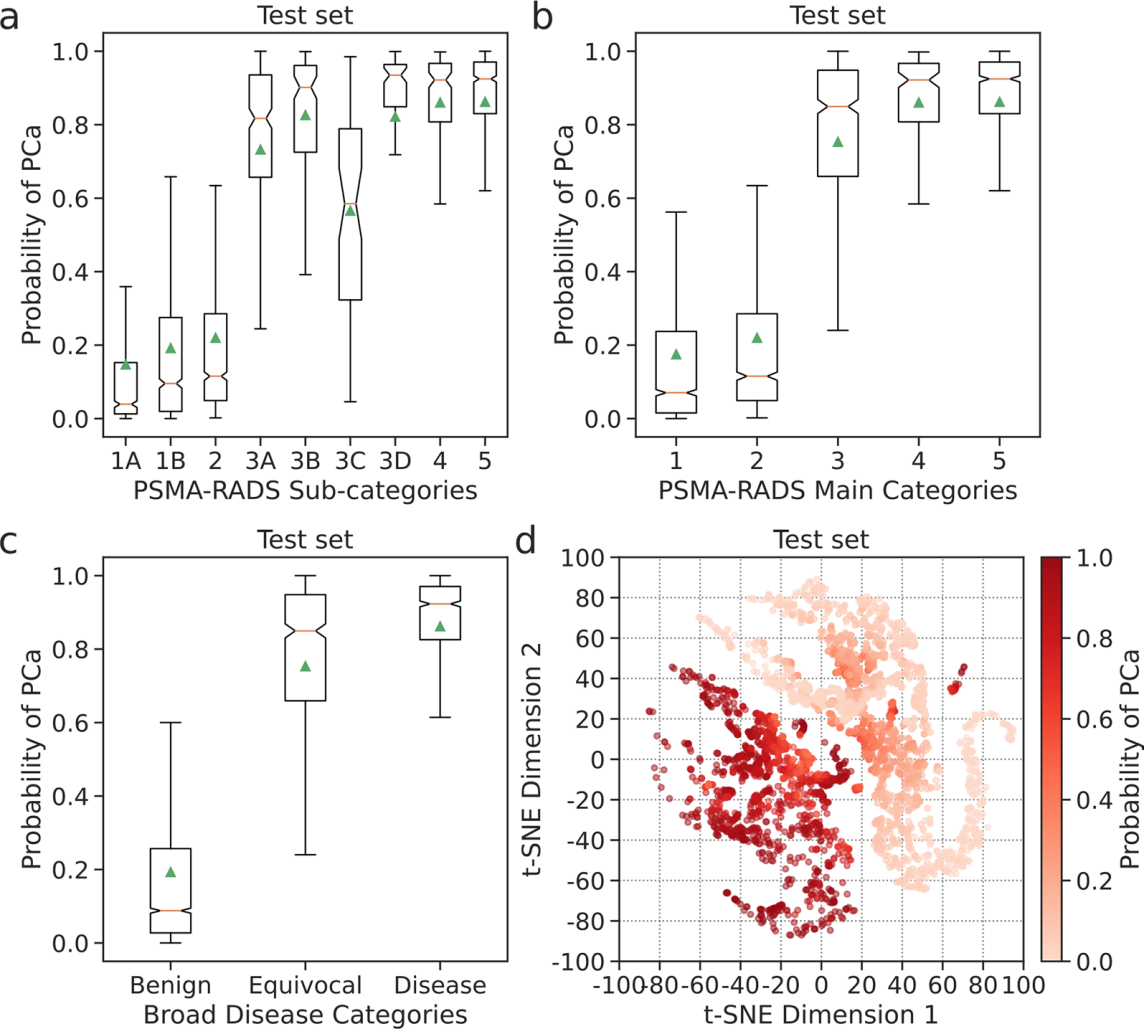
Notched boxplots of probability scores according to each of the PSMA-RADS sub-categories (**a**), the main PSMA-RADS categories (**b**), and the broad disease categories (**c**) where the green triangles correspond to the mean and the horizontal lines correspond to the median. Probability scores that reflect the likelihood of PCa were depicted on a t-SNE scatter plot (**d**)

Boxplots of probability scores reflecting the likelihood of PCa are shown in Fig. 6a–c. Higher probability scores were assigned to lesions with higher PSMA-RADS scores (Fig. 6b). PSMA-RADS-1 and -2 lesions had a mean probability score of 0.19 corresponding to benign findings (Fig. 6c). PSMA-RADS-4 and -5 lesions had a mean probability score of 0.86, reflecting the high likelihood of PCa. PSMA-RADS-3 lesions had an intermediate mean probability score of 0.75 corresponding to equivocal findings. However, PSMA-RADS-3C lesions had a significantly lower mean probability score of 0.57 (*P* < 0.05) when compared to PSMA-RADS-3A, -3B, and -3D lesions (Fig. 6a). This reflects the PSMA-RADS categorization scheme since PSMA-RADS-3C lesions are atypical for PCa [5]. The distribution of probability scores on t-SNE space (Fig. 6d) showed an increased likelihood of PCa from the benign to the disease clusters.

### Feature importance

**Fig. 7:**
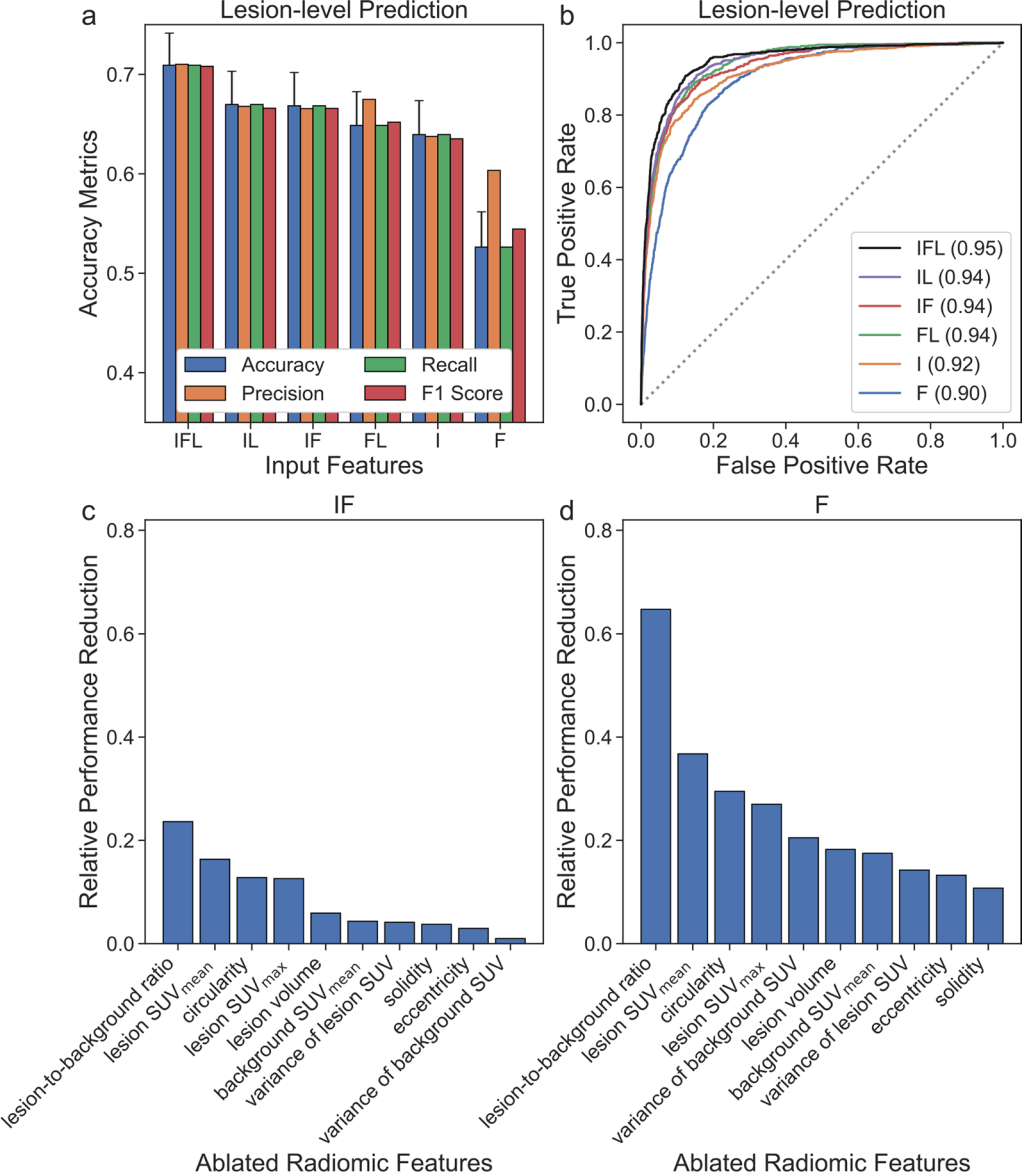
Accuracy metrics (**a**) and ROC curves (**b**) for different input feature combinations and the relative reduction in overall accuracy due to radiomic feature ablation (**c**, **d**). Error bars correspond to 95% confidence intervals (**a**)

The network trained on all input features had the highest performance across all evaluation metrics for lesion-level prediction (Fig. 7a, b and Additional file 1: Table S4) and had a significantly higher overall accuracy than all other networks (*P* < 0.05). The network trained on both the image and radiomic features outperformed the networks trained only on either the image or radiomic features, highlighting the synergy in combining the radiomic and CNN-extracted features. The networks trained on both the image and tissue type information and both the radiomic features and tissue type information outperformed the networks trained only on either the image or tissue type information, respectively, highlighting the importance of the tissue type information.

The relative reductions in performance due to radiomic feature ablation are shown in Fig. 7c, d. Ablation of the lesion-to-background ratio and the mean standardized uptake value (SUV_mean_) of the lesion resulted in the highest and second highest reductions in performance, respectively, for the network given both the image and radiomic features and the network given only radiomic features, highlighting the importance of those features. Circularity and maximum standardized uptake value (SUV_max_) of the lesion were the third and fourth most important features in both cases, respectively, followed by lesion volume as the fifth or sixth most important feature. This emphasizes the importance of accurate lesion delineation for reliable extraction of radiomic features reflecting such intensity and shape characteristics.

## Discussion

PSMA PET imaging has shown superior performance in the detection and staging of primary and metastatic PCa compared to conventional imaging modalities such as CT, MRI, and bone scan [3, 28, 29]. Our framework incorporated DL and radiomics to classify findings on [^18^F]DCFPyL PET scans. The framework classified findings on the test set into appropriate PSMA-RADS categories and yielded AUROC values of 0.87 and 0.90 for lesion-level and patient-level predictions, respectively. The framework provided broad PCa classification with AUROC values of 0.92 and 0.85 on the test set for lesion-level and patient-level predictions, respectively. A t-SNE analysis showed prediction clusters consistent with the PSMA-RADS categorization scheme. The framework provided confidence and probability scores reflecting the uncertainty and likelihood of PCa, respectively.

Lesion-level PSMA-RADS classification performance was comparable across the test and validation sets, except for PSMA-RADS-3D lesions which were largely misclassified as PSMA-RADS-3A lesions on the test set. Such cases of inaccuracy would not affect the recommendation suggested by the PSMA-RADS framework since further work-up or follow-up imaging would be required for PSMA-RADS-3A and -3D lesions [5]. Three out of six lesions incorrectly classified as PSMA-RADS-3D lesions on the test set were PSMA-RADS-1A lesions (Fig. 3b), likely because PSMA-RADS-3D lesions lack uptake on PSMA PET imaging despite representing potential malignancy on anatomic imaging [5]. Similarly, 8/9 lesions incorrectly classified as PSMA-RADS-3C lesions on the test set were PSMA-RADS-1B and -2 lesions (Fig. 3b). These observations reflect the complexity of the PSMA-RADS-3 designation.

The framework maintained an overall accuracy of 0.77 (41/53) on the test with both automatically and manually extracted inputs for the patient-level PSMA-RADS classification (Table 2), highlighting the robustness of the framework. Similarly, the framework yielded overall accuracies of 0.85 (621/732) and 0.89 (47/53) on the test set with automatically extracted inputs for lesion-level and patient-level broad PCa classification, respectively (Fig. 3d, Table 3).

A t-SNE analysis revealed learned local and global relationships between the PSMA-RADS categories and benign, equivocal, and disease findings (Fig. 4). The framework provided confidence and probability scores, which may help radiologists interpret the predicted outputs to make a more informed clinical diagnosis (Figs. 5, 6). A high level of uncertainty could serve as a flag for physicians to put less weight on the predicted output or to take a second look when determining diagnosis [30]. The confidence and probability scores may assist in better defining how patients should be treated when they appear to have limited volume recurrent or metastatic disease and are being considered for metastasis-directed therapy [31].

PSMA PET radiotracers have been observed to have physiologic uptake patterns and uptake in various benign bone pathologies, which may result in false-positive findings [3]. Benign findings were accounted for in our dataset where PSMA-RADS-1 and PSMA-RADS-2 findings corresponded to certainly or almost certainly benign regions of uptake [5]. For example, of the 898 regions of uptake in the bone, 33 (3.67%) were PSMA-RADS-1A findings, 15 (1.67%) were PSMA-RADS-1B, and 53 (5.90%) were PSMA-RADS-2 (Fig. 1b). Our framework was trained to differentiate regions of uptake corresponding to PCa and benign findings.

The tissue type information was found to be especially important in improving overall performance (Fig. 7a, b and Additional file 1: Tables S3–S4). Incorporating CT or MRI imaging may provide further anatomic information, especially for lesions with low uptake on the PET image [13]. For example, incorporating dynamic contrast-enhanced MRI may help improve the detection and characterization of skeletal metastases in patients with PCa [32, 33]. While performing textural analysis is challenging on PET due to limited spatial resolution, incorporating higher-order radiomic features, such as gray-level co-occurrence matrix, gray-level run-length matrix, and gray-level size zone matrix, from CT or MRI imaging, may help further improve performance [34].

The four most important radiomic features were lesion-to-background ratio, lesion SUV_mean_, circularity, and lesion SUV_max_ in feature ablation experiments (Fig. 7c, d). The variance of the background SUV was relatively important for the network given only radiomic features resulting in a 20.50% reduction in performance after ablation (Fig. 7d). However, the background SUV variance was the least important feature for the network that was given both the image and radiomic features with less than 1% reduction in performance (Fig. 7c), indicating that the CNN extracted important characteristics about the overall context of the lesion from the surrounding background in the input image. Interestingly, the network that was given both the image and the radiomic features was generally less sensitive to feature ablations and had smaller reductions in relative performance when compared to the network that was not given the image (Fig. 7c, d). This suggests that deep features extracted by the CNN are complementary to the radiomic features and can help compensate for the loss of information in ablated features.

While the highest PSMA-RADS score was used to determine the overall scan category, we also considered the impact of using the lower PSMA-RADS scores on patient-level performance which may be relevant in the case of less experienced readers. This was done by taking the median PSMA-RADS score predicted for individual lesions on the scan as the overall score compared to the maximum PSMA-RADS score. ROC curves and AUROC values for patient-level prediction when using manually extracted inputs are shown in Additional file 1: Fig. S6. As expected, using the maximum PSMA-RADS score yielded more reliable patient-level predictions than when using the median PSMA-RADS score, which yielded a lower AUROC value of 0.62 (Additional file 1: Fig. S6).

The framework’s performance was affected by the class imbalance present in the dataset (Fig. 1b). The PSMA-RADS-3C and -3D categories had the lowest performance and the fewest lesions in the entire dataset. Most scans had an overall PSMA-RADS score of either PSMA-RADS-4 or -5 further contributing to the patient-level class imbalance (Table 1). To combat class imbalances, generative adversarial networks could be leveraged to generate a large amount of simulated data to train the framework [35–37]. Training the framework using ensemble learning may also improve performance as such meta-learning approaches have had success for classification and prognostic tasks [38, 39].

Related work by Johnsson et al. introduced aPROMISE, a software platform for lesion detection in PSMA PET/CT [13]. In aPROMISE, U-net-based anatomical segmentations of bones and organs on the CT image are fused to the PET image, and lesion detection and segmentation are performed by blob detection and fast marching method, respectively [13]. In contrast, our approach performs deep learning-based lesion classification and segmentation using only the PET image. Another key difference is that aPROMISE is based on the PROMISE criteria, whereas our approach is based on PSMA-RADS [5, 13].

Our study had limitations. First, the framework was validated with physician-annotated PSMA-RADS categories subject to inter-operator variability. While the PSMA-RADS categorization scheme has been shown to have a high inter-observer agreement rate, further validation of the framework by histopathology or a multiple-reader consensus study is important for clinical translation [17, 18, 40]. Second, the framework was trained on a per-slice basis. Incorporating the whole imaged volume may help provide anatomic context by considering the presence of other lesions, for example, in the chest or abdomen regions [41, 42]. Third, while the framework incorporates lesion classification and segmentation tasks, the framework does not perform lesion detection. Incorporating the automated detection task could help identify regions of uptake that might be missed [43, 44].

## Conclusion

In conclusion, a DL- and radiomics-based framework was developed and performed lesion-level and patient-level PSMA-RADS and PCa classification on PSMA PET images. A t-SNE analysis revealed learned relationships between the PSMA-RADS categories and disease findings on PSMA PET scans. The framework was interpretable and provided well-calibrated confidence and probability scores for each prediction.

## Supplementary Information

**Additional file 1.** Supplementary material.

## Data Availability

The datasets used and/or analyzed during the current study are available from the corresponding author upon reasonable request.

## Abbreviations

AUROC: Area under the receiver operating characteristic
CNN: Convolutional neural network
CT: Computed tomography
DL: Deep learning
MRI: Magnetic resonance imaging
PCa: Prostate cancer
PET: Positron emission tomography
PSMA: Prostate-specific membrane antigen
PSMA-RADS: Prostate-specific membrane antigen reporting and data systems
ROC: Receiver operating characteristic
ROI: Region of interest
SUV: Standardized uptake value
t-SNE: T-distributed stochastic neighbor embedding

## References

[1] Siegel RL, Miller KD, Fuchs HE, Jemal A. Cancer statistics, 2022. CA Cancer J Clin. 2022;72(1):7–33. 10.3322/caac.21708 35020204

[2] Maurer T, Eiber M, Schwaiger M, Gschwend JE (2016). Current use of PSMA–PET in prostate cancer management. Nat Rev Urol.

[3] Sheikhbahaei S, Afshar-Oromieh A, Eiber M, Solnes LB, Javadi MS, Ross AE (2017). Pearls and pitfalls in clinical interpretation of prostate-specific membrane antigen (PSMA)-targeted PET imaging. Eur J Nucl Med Mol Imaging.

[4] Sheikhbahaei S, Werner RA, Solnes LB, Pienta KJ, Pomper MG, Gorin MA, et al. Prostate-specific membrane antigen (PSMA)-targeted PET imaging of prostate cancer: an update on important pitfalls. Semin Nucl Med. 2019;49(4):255–70. 10.1053/j.semnuclmed.2019.02.006 31227049

[5] Rowe SP, Pienta KJ, Pomper MG, Gorin MA (2018). PSMA-RADS version 1.0: a step towards standardizing the interpretation and reporting of PSMA-targeted PET imaging studies. Eur Urol.

[6] Reyes DK, Demehri S, Werner RA, Pomper MG, Gorin MA, Rowe SP (2019). PSMA-targeted [18F] DCFPyL PET/CT-avid lesions in a patient with prostate cancer: Clinical decision-making informed by the PSMA-RADS interpretive framework. Urol Case Reports.

[7] Hosny A, Parmar C, Quackenbush J, Schwartz LH, Aerts HJWL (2018). Artificial intelligence in radiology. Nat Rev Cancer.

[8] Vial A, Stirling D, Field M, Ros M, Ritz C, Carolan M (2018). The role of deep learning and radiomic feature extraction in cancer-specific predictive modelling: a review. Transl Cancer Res.

[9] Hatt M, Krizsan AK, Rahmim A, Bradshaw TJ, Costa PF, Forgacs A, et al. Joint EANM/SNMMI guideline on radiomics in nuclear medicine. Eur J Nucl Med Mol Imaging. 2022;1–24. 10.1007/s00259-022-06001-6 PMC9816255 36326868

[10] Cysouw MCF, Jansen BHE, van de Brug T, Oprea-Lager DE, Pfaehler E, de Vries BM, et al. Machine learning-based analysis of [18F] DCFPyL PET radiomics for risk stratification in primary prostate cancer. Eur J Nucl Med Mol Imaging. 2020;1–10. 10.1007/s00259-020-04971-z PMC7835295 32737518

[11] Pozaruk A, Pawar K, Li S, Carey A, Cheng J, Sudarshan VP, et al. Augmented deep learning model for improved quantitative accuracy of MR-based PET attenuation correction in PSMA PET-MRI prostate imaging. Eur J Nucl Med Mol Imaging. 2020;48(1):9–20. 10.1007/s00259-020-04816-9 32394162

[12] Erle A, Moazemi S, Lütje S, Essler M, Schultz T, Bundschuh RA (2021). Evaluating a machine learning tool for the classification of pathological uptake in whole-body PSMA-PET-CT scans. Tomogr Multidiscip.

[13] Johnsson K, Brynolfsson J, Sahlstedt H, Nickols NG, Rettig M, Probst S, et al. Analytical performance of aPROMISE: automated anatomic contextualization, detection, and quantification of [18F] DCFPyL (PSMA) imaging for standardized reporting. Eur J Nucl Med Mol Imaging. 2021;49(3):1041–51. 10.1007/s00259-021-05497-8 PMC8803714 34463809

[14] Fan F-L, Xiong J, Li M, Wang G. On interpretability of artificial neural networks: a survey. IEEE Trans Radiat Plasma Med Sci. 2021. 10.1109/trpms.2021.3066428 PMC9105427 35573928

[15] Guo C, Pleiss G, Sun Y, Weinberger KQ. On calibration of modern neural networks. In: International conference on machine learning. PMLR. 2017. p. 1321–30.

[16] Ashrafinia S, Sadaghiani MS, Dalaie P, Tulbah R, Yin Y, Leung K (2019). Characterization of Segmented 18F-DCFPyL PET/CT Lesions in the Context of PSMA-RADS Structured Reporting. J Nucl Med. Soc Nuclear Med.

[17] Werner RA, Bundschuh RA, Bundschuh L, Javadi MS, Leal JP, Higuchi T (2018). Interobserver agreement for the standardized reporting system PSMA-RADS 1.0 on 18F-DCFPyL PET/CT Imaging. J Nucl Med. Soc Nuclear Med.

[18] Demirci E, Akyel R, Caner B, Alan-Selçuk N, Güven-Meşe Ş, Ocak M (2020). Interobserver and intraobserver agreement on prostate-specific membrane antigen PET/CT images according to the miTNM and PSMA-RADS criteria. Nucl Med Commun.

[19] Zhang M, Leung KH, Ma Z, Wen J, Avinash G. A Generalized approach to determine confident samples for deep neural networks on unseen data. Uncertain Safe Util Mach Learn Med Imaging Clin Image Based Proced. 2019;11840:65–74. 10.1007/978-3-030-32689-0_15 PMC6938161 31893285

[20] Leung KH, Marashdeh W, Wray R, Ashrafinia S, Pomper MG, Rahmim A, et al. A physics-guided modular deep-learning based automated framework for tumor segmentation in PET. Phys Med Biol. 2020;65(24):1–18. 10.1088/1361-6560/ab8535 PMC12243949 32235059

[21] Leung K, Ashrafinia S, Sadaghiani MS, Dalaie P, Tulbah R, Yin Y (2019). A fully automated deep-learning based method for lesion segmentation in 18F-DCFPyL PSMA PET images of patients with prostate cancer. J Nucl Med Soc Nuclear Med.

[22] Leung K, Marashdeh W, Wray R, Ashrafinia S, Rahmim A, Pomper M (2018). A deep-learning-based fully automated segmentation approach to delineate tumors in FDG-PET images of patients with lung cancer. J Nucl Med. Soc Nuclear Med.

[23] Rodríguez P, Bautista MA, Gonzalez J, Escalera S (2018). Beyond one-hot encoding: Lower dimensional target embedding. Image Vis Comput.

[24] AUEB MTRC. One-vs-each approximation to softmax for scalable estimation of probabilities. Adv Neural Inf Process Syst. 2016;4161–9.

[25] Kingma D, Ba J. Adam: A method for stochastic optimization. In: International conference on learning representations. 2014.

[26] Sheng VS, Zhang J, Gu B, Wu X (2017). Majority voting and pairing with multiple noisy labeling. IEEE Trans Knowl Data Eng IEEE.

[27] Kobak D, Berens P (2019). The art of using t-SNE for single-cell transcriptomics. Nat Commun.

[28] Rowe SP, Macura KJ, Mena E, Blackford AL, Nadal R, Antonarakis ES (2016). PSMA-based [18 F] DCFPyL PET/CT is superior to conventional imaging for lesion detection in patients with metastatic prostate cancer. Mol Imaging Biol.

[29] Maurer T, Gschwend JE, Rauscher I, Souvatzoglou M, Haller B, Weirich G (2016). Diagnostic efficacy of 68gallium-PSMA positron emission tomography compared to conventional imaging for lymph node staging of 130 consecutive patients with intermediate to high risk prostate cancer. J Urol.

[30] Kompa B, Snoek J, Beam AL (2021). Second opinion needed: communicating uncertainty in medical machine learning. NPJ Digit Med.

[31] Phillips R, Shi WY, Deek M, Radwan N, Lim SJ, Antonarakis ES (2020). Outcomes of observation vs stereotactic ablative radiation for oligometastatic prostate cancer: the ORIOLE phase 2 randomized clinical trial. JAMA Oncol.

[32] Zhao J, Kader A, Mangarova DB, Brangsch J, Brenner W, Hamm B (2021). Dynamic contrast-enhanced MRI of prostate lesions of simultaneous [68Ga] Ga-PSMA-11 PET/MRI: comparison between intraprostatic lesions and correlation between perfusion parameters. Cancers (Basel).

[33] Kayhan A, Yang C, Soylu FN, Lakadamyalı H, Sethi I, Karczmar G (2011). Dynamic contrast-enhanced MR imaging findings of bone metastasis in patients with prostate cancer. World J Radiol..

[34] Rizzo S, Botta F, Raimondi S, Origgi D, Fanciullo C, Morganti AG (2018). Radiomics: the facts and the challenges of image analysis. Eur Radiol Exp.

[35] Kazuhiro K, Werner RA, Toriumi F, Javadi MS, Pomper MG, Solnes LB (2018). Generative adversarial networks for the creation of realistic artificial brain magnetic resonance Images. Tomography.

[36] Leung K, Rowe S, Shao W, Coughlin J, Pomper M, Du Y. Progressively growing GANs for realistic synthetic brain MR images. Soc Nuclear Med. 2021;62(1):1191.

[37] Shao W, Leung KH, Xu J, Coughlin JM, Pomper MG, Du Y (2022). Generation of digital brain phantom for machine learning application of dopamine transporter radionuclide imaging. Diagnostics.

[38] Xiao Y, Wu J, Lin Z, Zhao X (2018). A deep learning-based multi-model ensemble method for cancer prediction. Comput Methods Programs Biomed.

[39] Leung KH, Rowe SP, Pomper MG, Du Y (2021). A three-stage, deep learning, ensemble approach for prognosis in patients with Parkinson’s disease. EJNMMI Res.

[40] Giesel FL, Hadaschik B, Cardinale J, Radtke J, Vinsensia M, Lehnert W (2017). F-18 labelled PSMA-1007: biodistribution, radiation dosimetry and histopathological validation of tumor lesions in prostate cancer patients. Eur J Nucl Med Mol Imaging.

[41] Capobianco N, Sibille L, Chantadisai M, Gafita A, Langbein T, Platsch G (2022). Whole-body uptake classification and prostate cancer staging in 68Ga-PSMA-11 PET/CT using dual-tracer learning. Eur J Nucl Med Mol Imaging.

[42] Leung K, Sadaghiani MS, Dalaie P, Tulbah R, Yin Y, VanDenBerg R (2020). A deep learning-based approach for lesion classification in 3D 18F-DCFPyL PSMA PET images of patients with prostate cancer. J Nucl Med Soc Nuclear Med.

[43] Seetharaman A, Bhattacharya I, Chen LC, Kunder CA, Shao W, Soerensen SJC, et al. Automated detection of aggressive and indolent prostate cancer on magnetic resonance imaging. Med Phys. 2021;48(6):2960–72. 10.1002/mp.14855 PMC8360053 33760269

[44] Trägårdh E, Enqvist O, Ulén J, Hvittfeldt E, Garpered S, Belal SL, et al. Freely available artificial intelligence for pelvic lymph node metastases in PSMA PET-CT that performs on par with nuclear medicine physicians. Eur J Nucl Med Mol Imaging. 2022;49(10):3412–18. 10.1007/s00259-022-05806-9 PMC9308591 35475912

