# Supplementary material for: Deep learning and radiomics framework for PSMA-RADS classification of prostate cancer on PSMA PET
Source: EJNMMI Res. 2022 Dec 29;12:76. doi: 10.1186/s13550-022-00948-1 (PMC9800682; doi:10.1186/s13550-022-00948-1)
Supplement: Supplementary file 1 — Additional file 1. Supplementary material. [file 13550_2022_948_MOESM1_ESM.pdf]

Deep learning and radiomics framework for PSMA-RADS classification of prostate cancer on PSMA PET

Kevin H. Leung[1,2], Steven P. Rowe[2,3], Jeffrey P. Leal[2], Saeed Ashrafinia[2], Mohammad S. Sadaghiani[2], Hyun Woo Chung[4], Pejman Dalaie[2], Rima Tulbah[2], Yafu Yin[5], Ryan VanDenBerg[2], Rudolf A. Werner[6], Kenneth J. Pienta[3], Michael A. Gorin[7], Yong Du[2], Martin G. Pomper[1,2,3]

1. Department of Biomedical Engineering, Johns Hopkins University School of Medicine, Baltimore, MD, USA
2. The Russell H. Morgan Department of Radiology and Radiological Science, Johns Hopkins University School of Medicine, Baltimore, MD, USA
3. The James Buchanan Brady Urological Institute and Department of Urology, Johns Hopkins University School of Medicine, Baltimore, MD, USA
4. Department of Nuclear Medicine, Konkuk University Medical Center, Konkuk University School of Medicine, Seoul, Korea
5. Department of Nuclear Medicine, Xinhua Hospital, Shanghai Jiao Tong University School of Medicine, Shanghai, China
6. Department of Nuclear Medicine, University Hospital Würzburg, Würzburg, Germany
7. The Milton and Carroll Petrie Department of Urology, Icahn School of Medicine at Mount Sinai, New York, NY, USA

Corresponding author: Kevin H. Leung, Email: kleung8@jhmi.edu.

Address: 601 N Caroline St. JHOC 4263, Baltimore, MD 21287, USA.

EJNMMI Research

SUPPLEMENTARY INFORMATION

CNN architecture and image feature extraction

The CNN architecture is shown in Figure 2b. A rectified linear unit (ReLU) activation function and batch normalization were applied after each convolutional layer. Spatial dropout was applied after all convolutional layers during training to regularize the network and prevent co-adaptation between hidden neurons. The first two convolutional blocks had two convolutional layers followed by a max-pooling layer, and the last two convolutional blocks had three convolutional layers followed by a max-pooling layer. Dropout probabilities of 0.1 and 0.25 were applied to the first and last two convolutional layer blocks, respectively. The cropped PET images were resampled with a nearest neighbor's interpolation yielding an image size of 64 × 64. While resampling the cropped image changes the relative lesion size, information about the lesion volume, measured in cubic centimeters (cc), was included in the radiomic feature set.

The crop size was determined by a bounding box ROI placed around each lesion on a per-slice basis in the axial view. Bounding boxes with diagonal lengths scaled by the lesion diameter by a factor of 1.0, 2.0, 3.0, 5.0, 7.5, 10.0, and the full field-of-view (FOV) were investigated to find the optimal ROI size (Figure S1a). The crop size defined by the bounding box with a diagonal length of 7.5 times the lesion diameter yielded the highest overall accuracy on the hold-out set (Figure S1c). The optimal bounding box size also significantly outperformed the networks trained on cropped images with bounding boxes using a diagonal length of 1.0, 2.0, 3.0, and a full FOV ($P < 0.05$).

U-net architecture and radiomic feature extraction

The U-net architecture is shown in Figure 2c. The U-net received only the cropped PET image containing a lesion as input and output the predicted lesion ROI. The encoder blocks of the U-net consisted of separable convolutional layers followed by max-pooling layers.

Transposed convolutional layers followed by up-sampling layers were used in the decoder blocks. Residual skip connections via addition were used after each convolutional block to pass information learned from upstream layers. A ReLU activation, batch normalization, and spatial dropout were applied after each convolutional layer in the U-net. A sigmoid activation function was used after the last output layer to yield the segmented output. The U-net was trained using the training set manual segmentations to automatically delineate the lesion on the cropped PET image (Figures S2–S3 and Table S1). The U-net was trained by optimizing a class-weighted binary cross-entropy loss function with the adaptive moment estimation stochastic optimization algorithm, Adam, using a batch size of 512 samples for 500 epochs.

The performance of the U-net was on the validation and test set evaluated on the basis of Dice similarity coefficient (DSC) to quantify the overlap between the predicted and manual segmentations. The U-net yielded a mean DSC of 0.84 (95% CI: 0.84, 0.85) and 0.83 (95% CI: 0.82, 0.83) on the validation and test sets, respectively, indicating accurate delineation. The distribution of DSC values for lesions in each PSMA-RADS category is shown on boxplots in Figure S2 and Table S1. Examples of predicted lesion ROIs compared to the manually segmented ROIs are shown in Figure S3.

Radiomic features were extracted from the lesion ROIs on a per-slice basis to capture lesion intensity and morphology characteristics (Figure S1b). Intensity characteristics included the mean and variance of lesion standardized uptake value (SUV) and background SUV, lesion-to-background ratio, and the maximum standardized uptake value (SUV_{max}) within the lesion. Morphological features included lesion volume, circularity, solidity, and eccentricity measures. A circular ROI around the lesion captured the background pixels (Figure S1b). Radiomic features extracted using ROIs with diameters scaled by the lesion diameter by a factor of 1.0, 2.0, 3.0, 5.0, 7.5, and 10.0 were investigated to find the optimal ROI diameter (Figure S1b). A fully connected network was trained using the radiomic features extracted with the varying circular

ROI sizes. The circular ROI with a diameter 3.0 times the lesion diameter yielded the highest overall accuracy (Figure S1d) on the hold-out set. The network trained on the radiomic features extracted from the optimal circular ROI significantly outperformed the networks trained using circular ROIs with diameters 1.0, 5.0, 7.5, and 10.0 times the lesion diameter ($P < 0.05$).

Tissue-type CNN classifier

The tissue-type CNN classifier architecture is shown in Figure 2b. The CNN received only the cropped PET image containing the lesion as input and output the predicted tissue type of the lesion. The tissue-type CNN was trained on a per-slice basis by optimizing a class-weighted categorical cross-entropy loss function with the Adam optimization algorithm using a batch size of 512 samples for 500 epochs. Evaluation metrics including overall accuracy, precision, recall, F1 score, receiver operating characteristic (ROC) curve, and area under ROC curve (AUROC) were assessed on the validation and test sets. The tissue-type CNN classifier yielded an overall accuracy of 0.82 (95% CI: 0.81, 0.83) and 0.77 (95% CI: 0.75, 0.78) and an AUROC value of 0.94 and 0.91 on the validation and test sets, respectively, indicating accurate tissue type classification (Figure S4 and Table S2).

Hyperparameter optimization and training

The hyperparameters of the framework, including batch size and the number of training epochs, were optimized via a grid search. The hyperparameter sweep for batch size was performed for 32, 64, 128, 256, and 512 samples. The hyperparameter sweep for the number of training epochs was performed for 200, 300, 400, 500, and 1000 epochs. Hold-out cross-validation was performed during the grid search hyperparameter optimization using a randomly partitioned hold-out set consisting of 15% of the training set. The final network architecture was trained with a batch size of 512 samples for 500 epochs on the training set with early stopping on the hold-out set.

SUPPLEMENTARY TABLES

Table S1: U-net performance on the basis of Dice similarity coefficient.

PSMA-RADS Category	Validation set	Test set
1A	0.84 (0.83, 0.86)	0.83 (0.82, 0.84)
1B	0.76 (0.74, 0.77)	0.75 (0.74, 0.76)
2	0.86 (0.85, 0.87)	0.85 (0.84, 0.86)
3A	0.88 (0.87, 0.90)	0.86 (0.85, 0.88)
3B	0.84 (0.81, 0.87)	0.87 (0.85, 0.89)
3C	0.80 (0.75, 0.85)	0.82 (0.79, 0.85)
3D	0.80 (0.72, 0.87)	0.88 (0.85, 0.90)
4	0.91 (0.90, 0.92)	0.88 (0.86, 0.90)
5	0.86 (0.85, 0.87)	0.85 (0.84, 0.86)
All Classes	0.84 (0.84, 0.85)	0.83 (0.82, 0.83)

Note.–Data in parenthesis correspond to 95% confidence intervals.

Table S2: Tissue-type CNN performance.

	**Validation set**			
Tissue Type	Precision	Recall	F1 Score	AUROC
Bone	0.76	0.77	0.76	0.93
Lymphadenopathy	0.90	0.89	0.90	0.97
Prostate	0.75	0.99	0.86	1.00
Soft Tissue	0.79	0.75	0.77	0.90
All Classes	0.82	0.82	0.82	0.95
	**Test set**			
Tissue Type	Precision	Recall	F1 Score	AUROC
Bone	0.51	0.42	0.46	0.84
Lymphadenopathy	0.89	0.85	0.87	0.94
Prostate	0.85	0.91	0.88	0.99
Soft Tissue	0.66	0.74	0.70	0.88
All Classes	0.77	0.77	0.77	0.93

Table S3: Input feature combinations.

Input Features	Description of Feature Combinations
IFL	PET Image, radiomic features, tissue type of the lesion
IL	PET Image, tissue type of the lesion
IF	PET Image, radiomic features
FL	Radiomic features, tissue type of the lesion
I	PET Image
F	Radiomic features

Table S4: Input feature importance.

	Lesion-level performance				
Input features	Accuracy	Precision	Recall	F1 Score	AUROC
IFL	0.71 (0.68, 0.74)	0.71	0.71	0.71	0.95
IL	0.67 (0.64, 0.70)	0.67	0.67	0.67	0.94
IF	0.67 (0.63, 0.70)	0.67	0.67	0.67	0.94
FL	0.65 (0.61, 0.68)	0.67	0.65	0.65	0.94
I	0.64 (0.61, 0.67)	0.64	0.64	0.64	0.92
F	0.53 (0.49, 0.56)	0.60	0.53	0.54	0.90

Note.–Data in parenthesis correspond to 95% confidence intervals.

SUPPLEMENTARY FIGURES

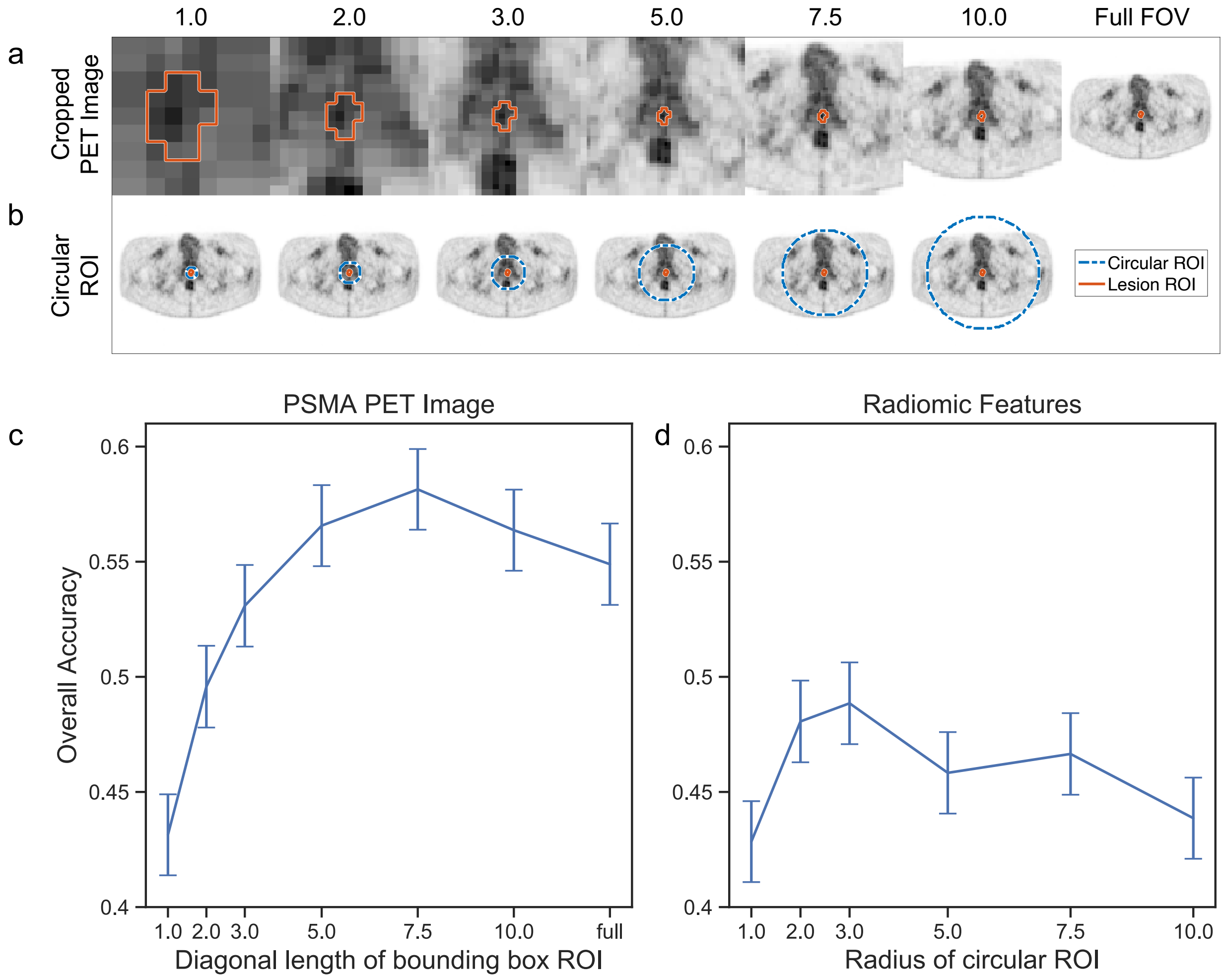

Fig. S1 Optimization of lesion crop size (a and c) and circular region of interest (ROI) size for extracting radiomic features (b and d). Error bars correspond to 95% confidence intervals (c–d).

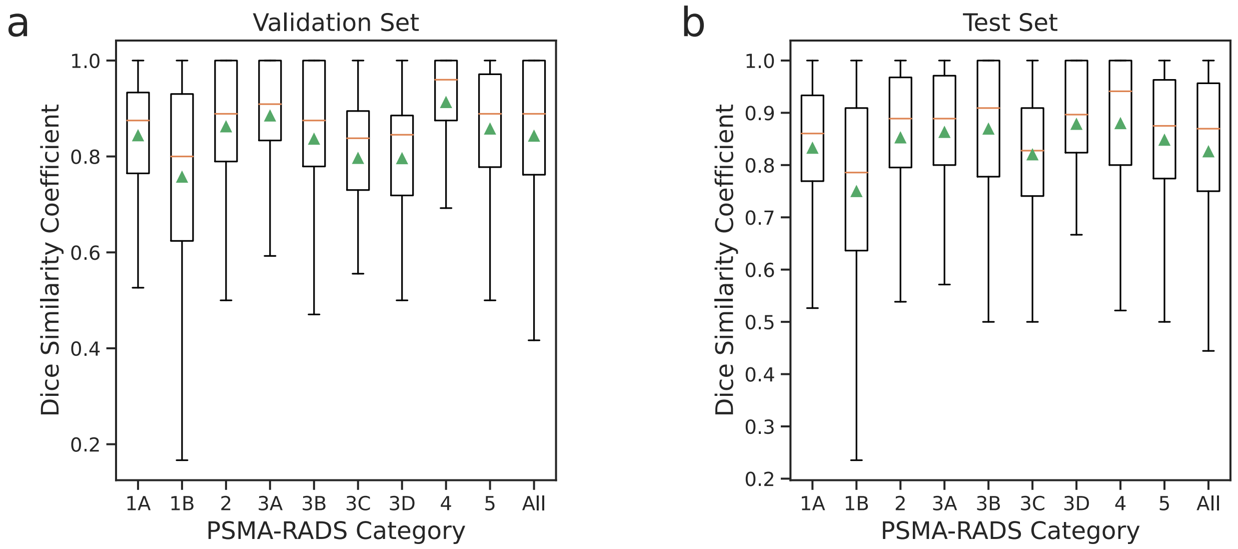

Fig. S2 U-net performance on the validation (a) and test sets (b).

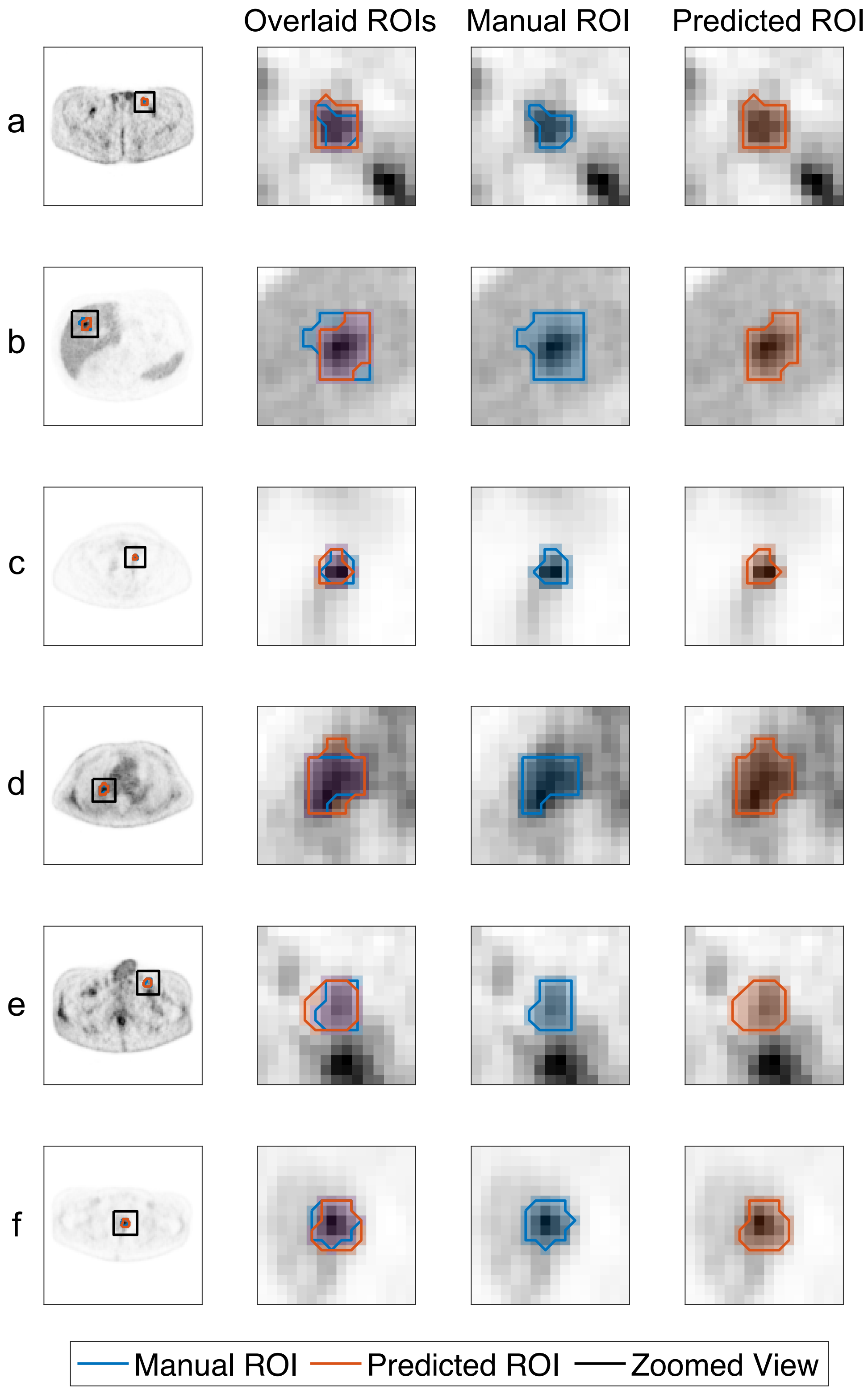

Fig. S3 Examples of predicted lesion ROIs by the U-net.

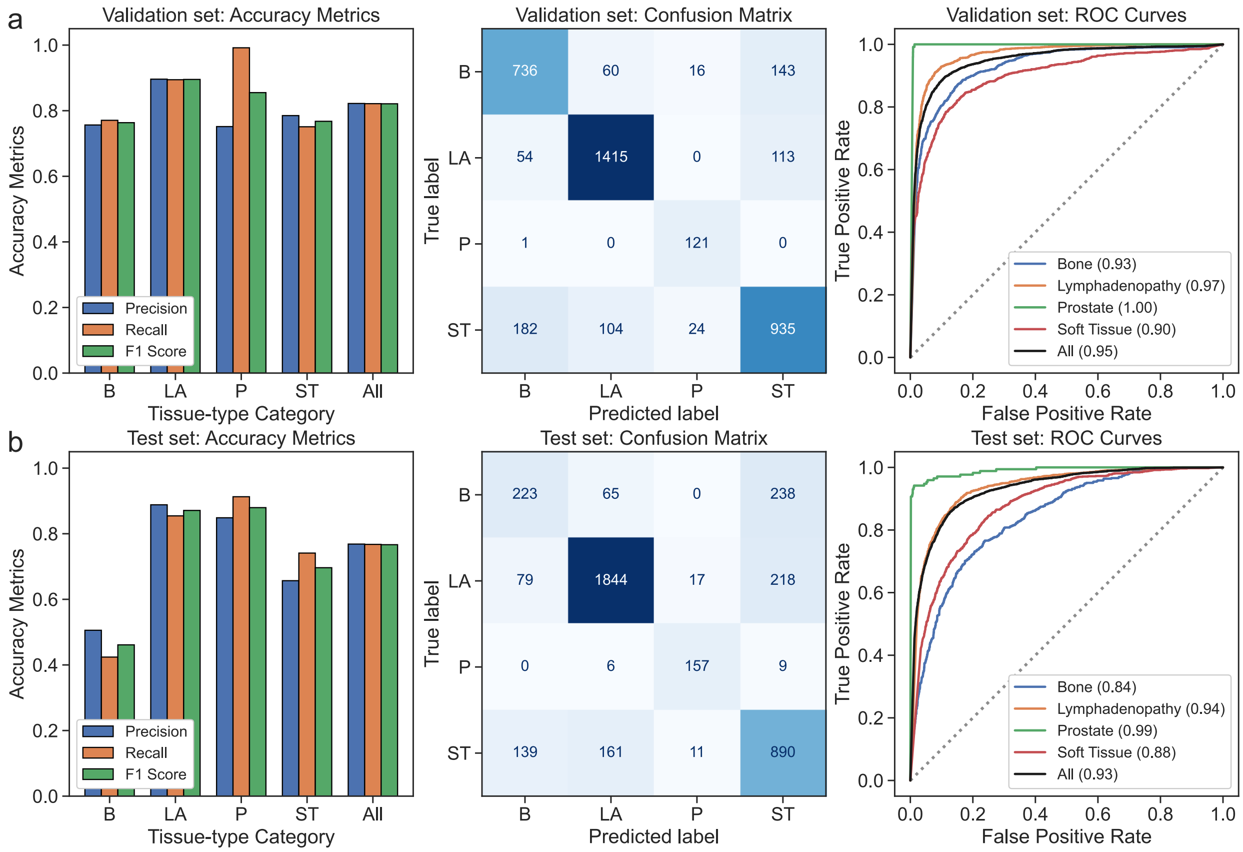

Fig. S4 Tissue-type CNN performance on the validation (a) and test sets (b). B = bone. LA = lymphadenopathy. P = prostate. ST = soft tissue.

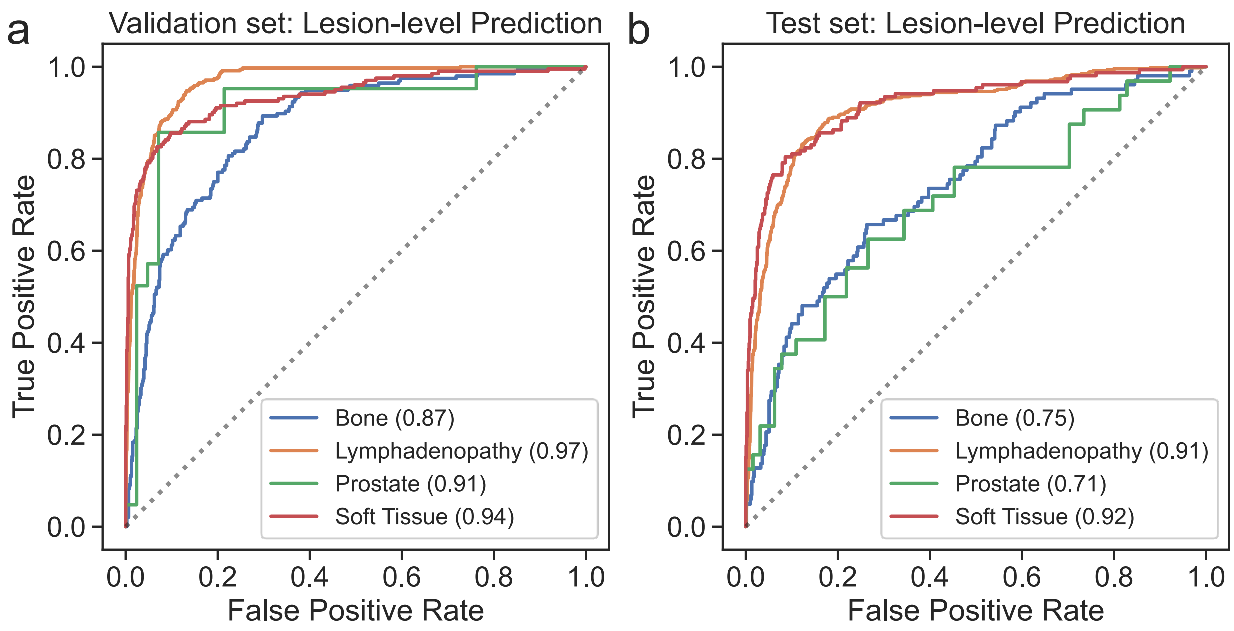

Fig. S5 ROC curves for lesion-level PSMA-RADS classification of lesions with different tissue types on the validation (a) and test (b) sets.

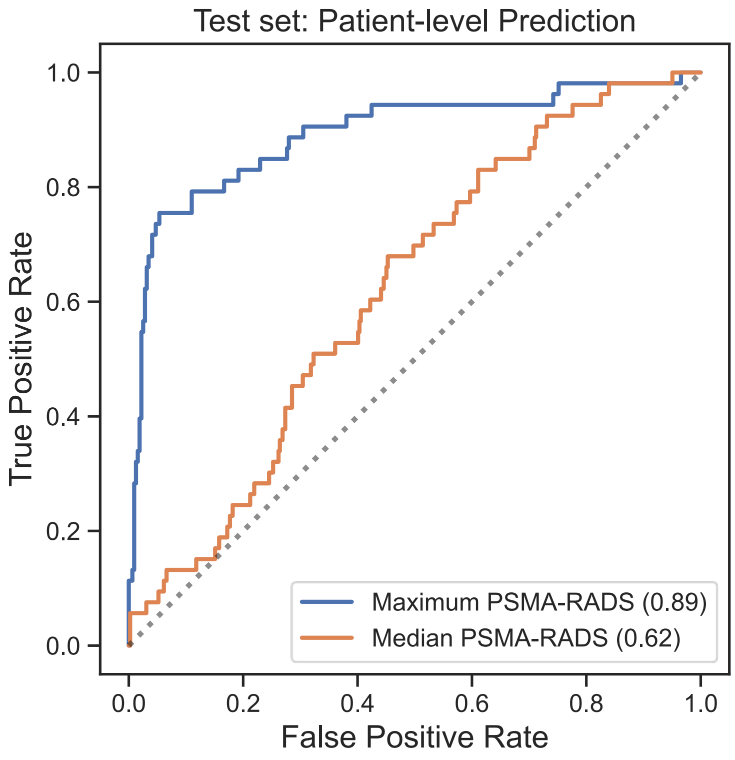

Fig. S6 ROC curves for patient-level prediction on the PSMA-RADS classification task when using the highest versus median PSMA-RADS overall scan scores.
